# A designer rice NLR immune receptor confers resistance to the rice blast fungus carrying noncorresponding avirulence effectors

**DOI:** 10.1073/pnas.2110751118

**Published:** 2021-10-26

**Authors:** Yang Liu, Xin Zhang, Guixin Yuan, Dongli Wang, Yangyang Zheng, Mengqi Ma, Liwei Guo, Vijai Bhadauria, You-Liang Peng, Junfeng Liu

**Affiliations:** ^a^State Key Laboratory of Agrobiotechnology, China Agricultural University, Beijing 100193, China;; ^b^Ministry of Agriculture Key Laboratory for Crop Pest Monitoring and Green Control, China Agricultural University, Beijing 100193, China;; ^c^Joint International Research Laboratory of Crop Molecular Breeding, China Agricultural University, Beijing 100193, China;; ^d^State Key Laboratory for Conservation and Utilization of Bio-Resources in Yunnan, Yunnan Agricultural University, Kunming 650201, China

**Keywords:** NLR immune receptor, integrated domain, recognition, multilines, broad spectrum resistance

## Abstract

Plant nucleotide-binding and leucine-rich repeat (NLR) receptors recognize avirulence effectors directly through their integrated domains (IDs) or indirectly via the effector-targeted proteins. Previous studies have succeeded in generating designer NLR receptors with new recognition profiles by engineering IDs or targeted proteins based on prior knowledge of their interactions with the effectors. However, it is yet a challenge to design a new plant receptor capable of recognizing effectors that function by unknown mechanisms. Several rice NLR immune receptors, including RGA5, possess an integrated heavy metal–associated (HMA) domain that recognizes corresponding *Magnaporthe oryzae* Avrs and ToxB-like (MAX) effectors in the rice blast fungus. Here, we report a designer rice NLR receptor RGA5^HMA2^ carrying an engineered, integrated HMA domain (RGA5-HMA2) that can recognize the noncorresponding MAX effector AvrPib and confers the RGA4-dependent resistance to the *M. oryzae* isolates expressing AvrPib, which originally triggers the Pib-mediated blast resistance via unknown mechanisms. The RGA5-HMA2 domain is contrived based on the high structural similarity of AvrPib with two MAX effectors, AVR-Pia and AVR1-CO39, recognized by cognate RGA5-HMA, the binding interface between AVR1-CO39 and RGA5-HMA, and the distinct surface charge of AvrPib and RAG5-HMA. This work demonstrates that rice NLR receptors with the HMA domain can be engineered to confer resistance to the *M. oryzae* isolates noncorresponding but structurally similar MAX effectors, which manifest cognate NLR receptor–mediated resistance with unknown mechanisms. Our study also provides a practical approach for developing rice multilines and broad race spectrum–resistant cultivars by introducing a series of engineered NLR receptors.

## Significance

In this study, we generated a mutant of the rice nucleotide-binding and leucine-rich repeat (NLR) immunity receptor RGA5 by engineering its heavy metal–associated domain that recognizes the noncorresponding *Magnaporthe oryzae* Avrs- and ToxB-like effector AvrPib and confers resistance in transgenic rice to the blast fungus isolates with AvrPib, which is known to trigger blast resistance in rice cultivars carrying the R gene *Pib*, albeit by unknown mechanisms. Thus, this work demonstrates that integrated domain-containing plant NLR receptors can be engineered to confer resistance to pathogens carrying avirulence effectors that trigger plant immunity by unknown mechanisms, thereby providing a practical approach for developing multilines and cultivars with broad race spectrum resistance.

Many gene-for-gene diseases, in which hosts and pathogens recognize each other by their cognate resistance (R) and avirulence (Avr) proteins, pose a serious threat to global food security. Managing such diseases is cumbersome as *R* gene–mediated resistance is frequently compromised by virulent races of the pathogens, which are evolved by losing the Avr function of corresponding Avr genes (1). To avert the resistance erosion, several strategies have been proposed, including the utilization of broad, race spectrum–resistant cultivars and multilines, each of which carries a distinct R protein capable of recognizing corresponding Avr proteins in the pathogens (2, 3). Multiline cultivars have been well demonstrated in managing stripe rust and the powdery mildew of wheat (4, 5). However, cultivars with broad race spectrum resistance are very limited, and breeding multilines is time-consuming, expensive, and laborious. The genetic engineering of *R* genes may address the challenges and accelerate the development of broad, race spectrum–resistant cultivars and multilines. Diverse types of *R* genes have been identified, one major group of which encodes nucleotide-binding and leucine-rich repeat (NLR) receptors (6). These NLRs are typically composed of an N-terminal coiled-coil (CC) or Toll/IL-1 receptor (TIR) domain, a central nucleotide-binding domain, and a C-terminal leucine-rich repeat domain. Interestingly, many NLR receptors contain an additional integrated domain (ID), such as the heavy metal–associated (HMA) domain or WRKY domain. These IDs determine the specificity of NLR receptors recognizing Avr effectors and thus have been considered as an ideal target for engineering new types of resistance (7–10).

As reported, the bacterial protease effector AvrPphB from *Pseudomonas syringae* cleaves the *Arabidopsis thaliana* PBS1 to activate the RPS5-dependent resistance (11). The substitution of the AvrPphB cleavage site in PBS1 with that of protease effector AvrRpt2, which originally activates RPS2-mediated resistance, or of tobacco etch virus protease Nia can enable transgenic plants to acquire the resistance to pathogens carrying noncorresponding Avr effectors (9, 12, 13). These examples are achieved based on the prior knowledge of molecular or structural biology on Avr effectors and their targeted IDs in the NLR receptors or targeted proteins that modulates the NLR receptor–mediated resistance. However, most plant NLR receptors lack an ID, or their regulatory proteins targeted by Avr effectors are unknown (14, 15). Thus, it is a challenge to design an NLR receptor that can recognize noncorresponding Avr effectors, which trigger their cognate R protein–mediated resistance by unknown mechanisms for application in improving crop disease resistance.

Rice blast caused by *Magnaporthe oryzae* is a typical gene-for-gene disease, which threatens rice production across the globe. So far, over 100 blast *R* genes have been mapped onto the rice genome, and more than 20 of them have been cloned, which are all known to encode NLR immune receptors, including *Pib*, *Piz-t*, *Pik*1, and *RGA*5 (16). The *M. oryzae* genome encodes a large number of *M. oryzae* Avrs- and ToxB-like (MAX) effectors, which are sequence-unrelated, but structurally similar, proteins (17–21) (*SI Appendix*, Fig. S1). AvrPib is an MAX effector known to trigger Pib-mediated blast resistance in rice, but no direct interaction has been verified between them, and thus, the recognition mechanism remains unclear (20, 22). AvrPiz-t is another MAX effector not directly interacting with the cognate NLR receptor Piz-t, although its virulence function has been established (21, 23, 24). Interestingly, *RGA5* and *Pik1* encode NLR immune receptors with a HMA domain, which acts as an ID directly binding corresponding MAX Avr effectors and mediates their helper NLR receptor–triggered immunity (17, 25). Pik-HMA specifically recognizes the effector AVR-Pik, while RGA5-HMA detects two sequence-unrelated effectors AVR-Pia and AVR1-CO39 (17, 26). Furthermore, Pik-HMA and RGA5-HMA are structurally similar, albeit they recognize distinct MAX effectors (17, 26, 27). A recent study showed that a designed HMA domain of the rice immunity receptor Pikp-1, with the crucial residues of the HMA domain from the Pikm1 allele, acquired the capacity of Pikm-HMA to recognize related MAX effectors AVR-PikA, AVR-PikD, and AVR-PikE of *M. oryzae*, thereby expanding the recognition profile of original Pikp-1 that only recognizes AVR-PikD (28, 29). In this study, we reasoned that it might be plausible to design rice NLR immune receptors with the capability to detect noncorresponding MAX effectors by genetically engineering the HMA domains, which will be useful for efficiently developing broad, race spectrum–resistant cultivars and multilines. To verify this hypothesis, we engineered the HMA domain of RGA5 by changing its interface with AVR1-CO39 and the K-rich region and obtained a version of the RGA5 NLR immune receptor capable of recognizing the noncorresponding MAX effector AvrPib. In particular, we verified that transgenic rice lines expressing the engineered RGA5 NLR immune receptor, with the helper NLR RGA4, could confer resistance to the *M. oryzae* isolates carrying functional AvrPib. Our findings demonstrate that rice NLR receptors with ID can be engineered to confer resistance to *M. oryzae* isolates with noncorresponding MAX effectors that trigger plant immunity by unknown mechanisms and provide a practical approach for breeding rice multilines and generating cultivars with broad race spectrum resistance to the blast disease.

## Results

### A Designed RGA5-HMA2 Can Interact with the Noncorresponding Effector AvrPib but Not with the Corresponding Effector AVR-Pia in Yeast Cells.

**Fig. 1.**
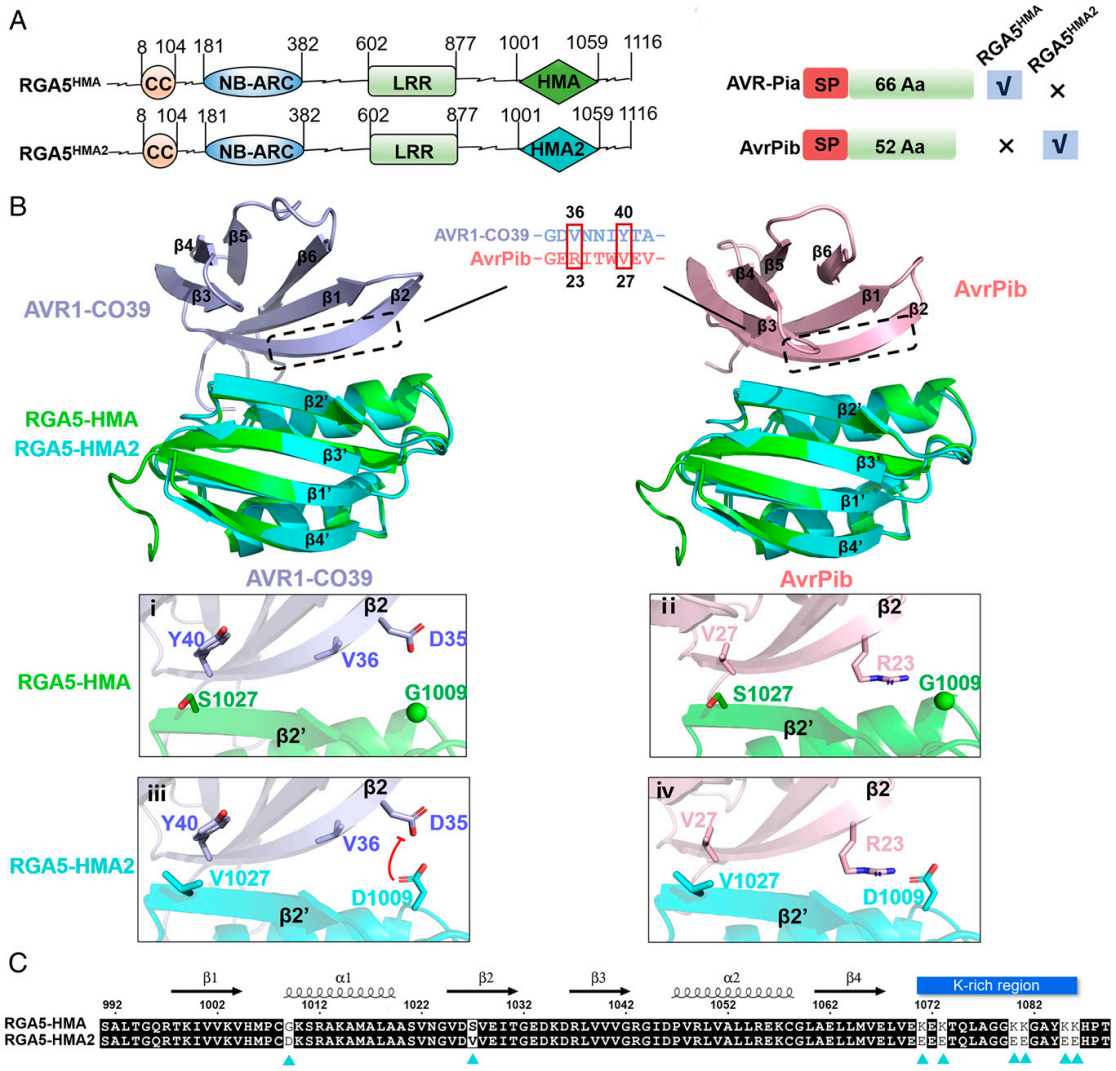
Engineering process of RGA5-HMA2 for recognizing the AvrPib. (*A*) A schematic showing structures of the wild-type RGA5 and the mutant RGA5^HMA2^. The wild-type HMA domain and the mutant HMA2 domain are shown in green and cyan, respectively. Features of AVR-Pia and AvrPib recognized by the wild-type RGA5 and the mutant RGA5^HMA2^ are shown on the right side. (*B*) The sequence alignment in the middle shows the β2 amino acid of AVR1-CO39 with AvrPib, and the magenta blocks show two key residues differing between AVR1-CO39 and AvrPib for interactions. The residues around the two key residues in the complex of AVR1-CO39/HMA (*i*), AvrPib/HMA (*ii*), AVR1-CO39/HMA2 (*iii*), and AvrPib/HMA2 (*iv*) are predicted by the superposition of the structures of HMA domains. (*C*) Amino acid sequence alignment showing mutations in RGA5-HMA2, as compared with the wild-type HMA. Secondary structural features of the two HMA domains are shown above of alignment, and mutations of RGA5-HMA enhancing the binding affinity to AvrPib and blocking interaction with AVR-Pia are indicated below by cyan triangles. Lys-rich domain is shown as a blue horizontal bar.

**Fig. 2.**
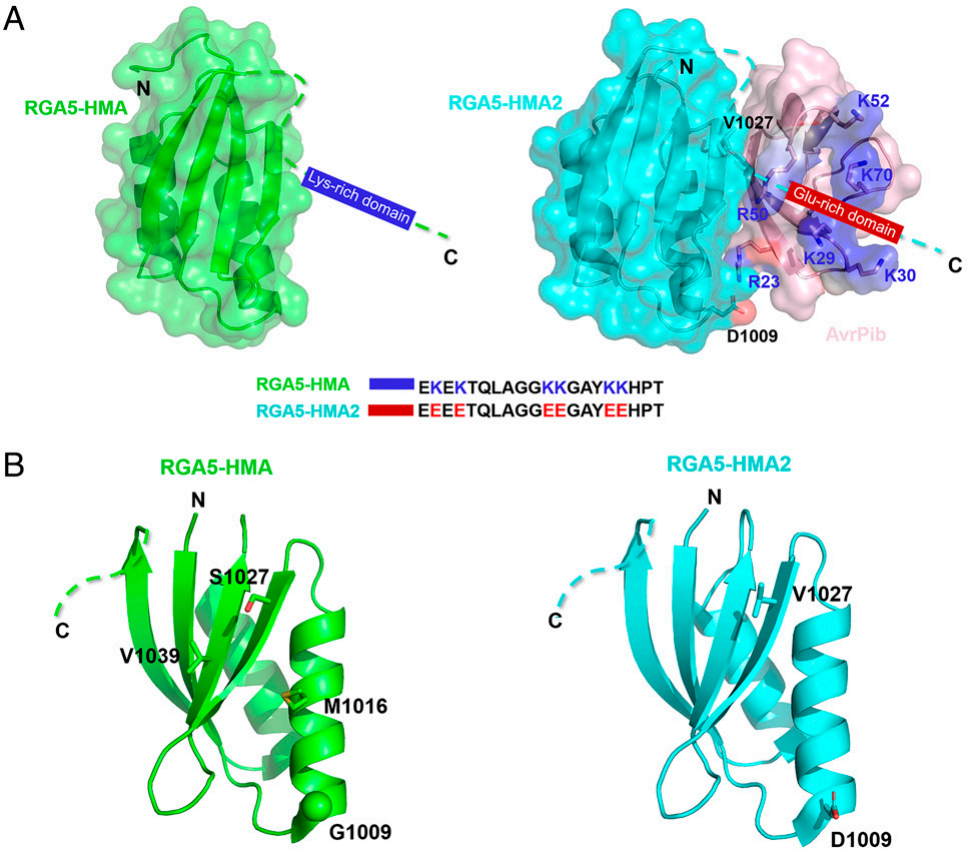
Comparison of the RGA5-HMA2 with RGA5-HMA structures. (*A*) Lysine residues in the K-rich region (between 1,070 and 1,090) of RGA5-HMA were replaced as glutamic acid to form an E-rich region in RGA5-HMA2 to increase the binding affinity with the positive patch of AvrPib (Protein Data Bank [PDB]: 5Z1V). The positive patch consisting of labeled Lys or Arg residues is shown in the blue surface of AvrPib. The C-terminal domains of RGA5-HMA2 and RGA5-HMA are aligned at the bottom. (*B*) Crystal structures of RGA5-HMA and RGA5-HMA2, and RGA5-HMA in green (PDB: 5ZNE) and RGA5-HMA2 in cyan.

**Fig. 3.**
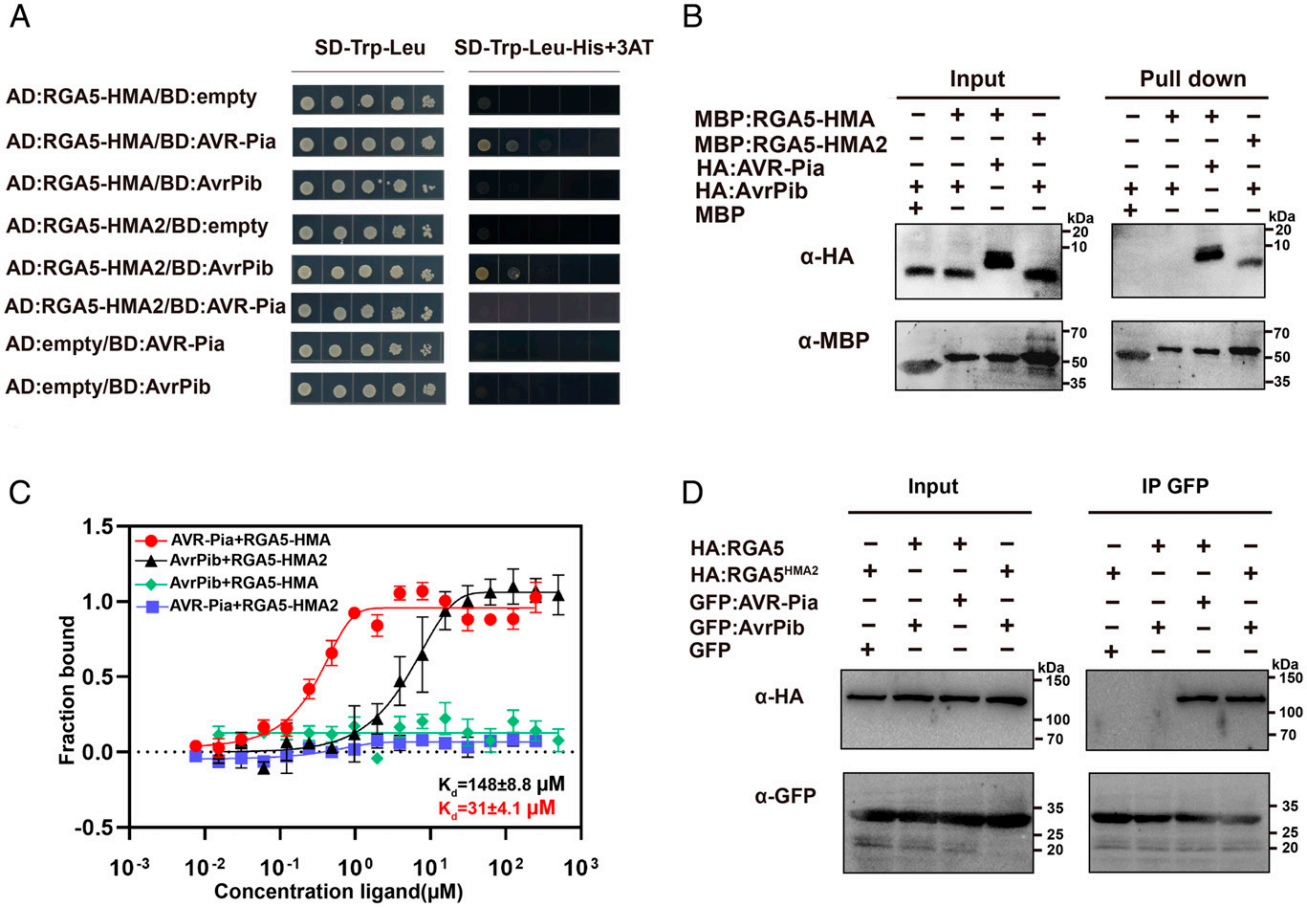
RGA5-HMA2 recognizes AvrPib in vitro and in vivo. (*A*) Y2H assays showing the specific interactions of RGA5-HMA domain (residues 982 to 1,116) with AVR-Pia and RGA5-HMA2 domain (residues 982 to 1,116) with AvrPib. (*B*) MBP pull-down assays showing the specific interaction of the RGA5-HMA domain with AVR-Pia and the RGA5-HMA2 domain with AvrPib. The recombinant proteins MBP-RGA5-HMA, MBP-RGA5-HMA2, HA-AvrPib, and HA-AVR-Pia purified from *E. coli* were used for the MBP pull-down analysis. The fusion proteins were detected using the anti-HA and anti-MBP antibodies. (*C*) MST analysis showing the dissociation constants of AvrPib and AVR-Pia with the RGA5-HMA or RGA5-HMA2 domain. The experiment was repeated three times. Bars ± SD (*n* = 3). (*D*) Co-IP of RGA5^HMA2^ (full-length RGA5 with the integrated engineered HMA domain [HMA2]) with AvrPib. *HA-RGA5^HMA2^* and *GFP-AvrPib* were transiently coexpressed in *N. benthamiana* leaves, and the proteins extracted from the leaves were incubated with GFP beads and detected separately by the anti-HA and anti-GFP antibodies.

As shown in Fig. 1 *A* and *B*, the rice NLR immune receptor RGA5 recognizes corresponding MAX effectors AVR1-CO39 and AVR-Pia by its HMA domain (25–27) (*SI Appendix*, Fig. S1). To design an RGA5-HMA domain with the capacity of recognizing the noncorresponding MAX effector AvrPib, we first compared the structures of AVR1-CO39 with that of AvrPib (20, 27). By the comparison, we found that the V36 and Y40 sites of AVR1-CO39 are located at the interface binding to RGA5-HMA, and the R23 and V27 residues of AvrPib likely correspond to the V36 and Y40 sites of AVR1-CO39 (Fig. 1*B*), suggesting that the R23 and V27 residues of AvrPib may be crucial to constructing an interface for interaction with an engineered RGA5-HMA. Therefore, we reasoned that the mutation causing two amino acid substitutions (S1027V and G1009D) in the interface of the RGA5-HMA domain might interact with AvrPib while decreasing the binding ability to AVR1-CO39 (Fig. 1*B*). V1027 of HMA2 could form the hydrophobic interaction with V27 of AvrPib, while it could affect the interaction with Y40 of AVR1-CO39. The G-to-D mutation at 1,009 of HMA2 could repel D35 of AVR1-CO39 while forming the salt bridge with R23 of AvrPib (Fig. 1*B*). To test this idea, we created an RGA5-HMA variant, RGA5-HMA^S1027V, G1009D^, and analyzed its interactions with another corresponding effector, AVR-Pia, and with the noncorresponding effector AvrPib by the yeast two-hybrid (Y2H) assays. As expected, the variant failed to interact with AVR-Pia; however, it also could not interact with AvrPib (*SI Appendix*, Fig. S2). Then, we shifted our focus to the K-rich region in the variable C terminus of RGA5-HMA (Fig. 1*B*). Since there is a positively charged patch on the surface of AvrPib (20), the C-terminal K-rich region of the HMA domain would have blocked the interaction between RGA5-HMA and AvrPib (Figs. 1*C* and 2*A*). To circumvent the issue, we generated the RGA5-HMA^K/E^ variant (K to E mutations including K at the 1,071, 1,073, 1,080, 1,081, 1,085, and 1,086 sites) (Fig. 2*A*). However, no interaction was detected between RGA5-HMA^K/E^ and AvrPib (*SI Appendix*, Fig. S2). Finally, we contrived another RGA variant, RGA5-HMA^S1027V, G1009D, K/E^ (henceforth RGA5-HMA2), by combining all the above mutations, which were confirmed to resemble the native RGA5-HMA structure (Fig. 2*B* and *SI Appendix*, Fig. S3). Again, we performed a Y2H assay, which showed that RGA5-HMA2 could interact with AvrPib but not with AVR-Pia (Fig. 3*A* and *SI Appendix*, Fig. S2 and Table S1), implying that the mutations in RGA5-HMA2, which do not affect the conformation of the domain, acquire the binding affinity to the originally unrecognized MAX effector AvrPib (Fig. 1*A* and *SI Appendix*, Fig. S3).

### Designer Receptor RGA5^HMA2^ with the RGA5-HMA2 Domain Can Interact with AvrPib In Planta.

To verify whether designer NLR RGA5^HMA2^ (i.e., full-length RGA5 carrying RGA5-HMA2) and RGA5-HMA2 (residues 982 to 1,116) can interact with AvrPib in planta, we performed maltose-binding protein (MBP) pull-down, microscale thermophoresis (MST), and coimmunoprecipitation (Co-IP) in addition to Y2H, in which RGA5-HMA2 or RGA5-HMA domain (residues 982 to 1,116) was applied (Fig. 3 *B*–*D*). In the pull-down assay, recombinant HA-AvrPib/MBP-RGA5-HMA2 and HA-AvrPib/MBP-RGA5-HMA proteins expressed in *Escherichia coli* showed that RGA5-HMA2 interacted with AvrPib in vitro (Fig. 3*B*). The data obtained from the MST assays also confirmed the interaction between RGA5-HMA2 and AvrPib, which displayed a dissociation constant (*K*_d_) of 148 μM, compared to 31 μM for the RGA5-HMA/AVR-Pia interaction. The interaction of RGA5-HMA2 with AvrPib, however, was a little weaker than that of the wild-type HMA (RGA5-HMA) with AVR-Pia (Fig. 3*C*). Further Co-IP assays showed that RGA5^HMA2^ but not RGA5 interacted with AvrPib in *Nicotiana benthamiana* (Fig. 3*D*). Taken together, the above data indicate that the designer NLR receptor RGA5^HMA2^ can specifically interact with AvrPib in plants via its engineered HMA domain, RGA5-HMA2 (*SI Appendix*, Table S1).

### RGA5^HMA2^ Recognizes AvrPib to Mediate RGA4-Triggered Cell Death in *N. benthamina* and *Oryza sativa*.

**Fig. 4.**
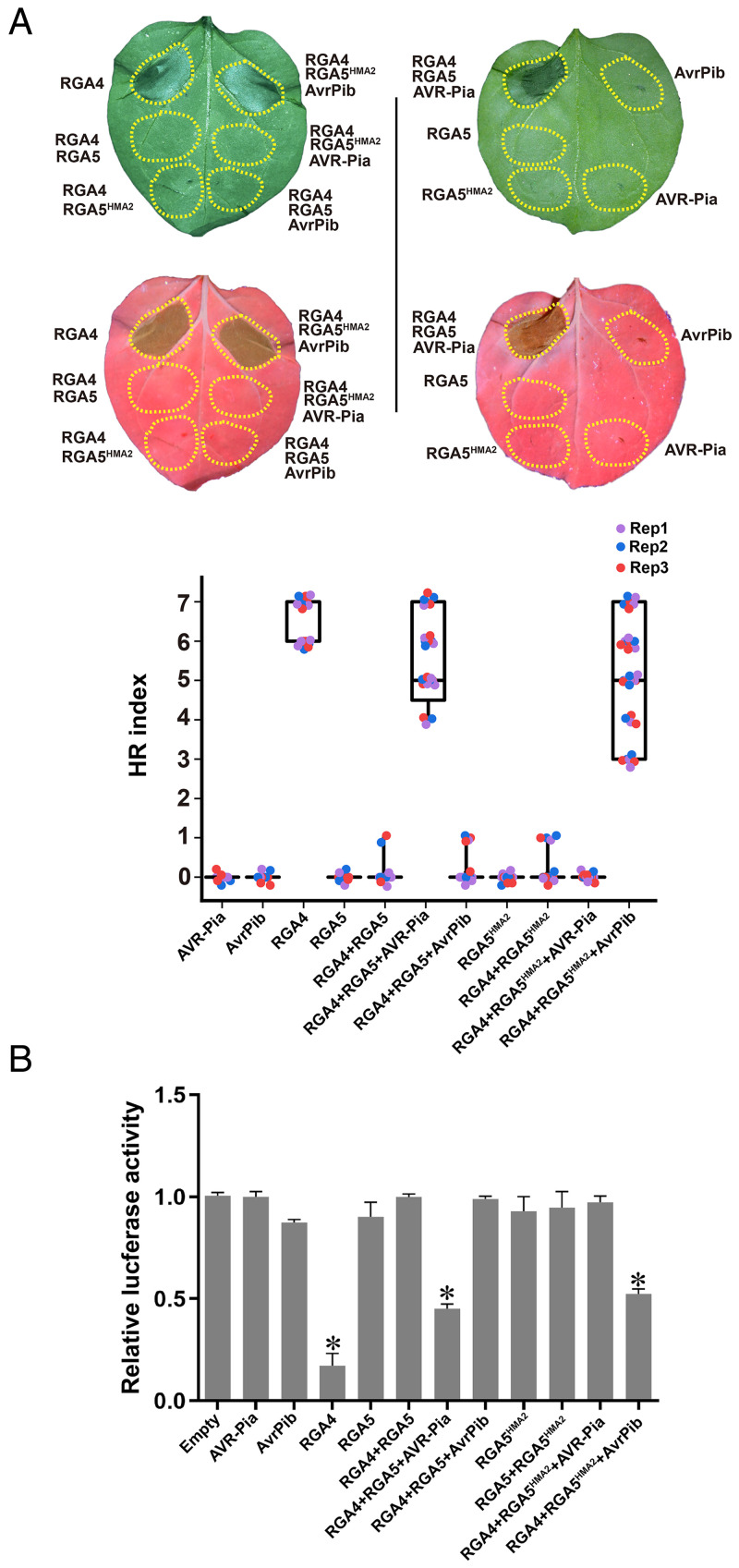
The interaction of AvrPib with RGA5^HMA2^ causes the RGA4-mediated plant cell death. (*A*) The representative leaf image of *N. benthamiana*. (*B*) The LUC activity in rice protoplasts showing plant cell death caused by the helper NLR receptor RGA4, RGA4/RGA5/AVR-Pia, and the RGA4/RGA5^HMA2^/AvrPib but not by RGA5, RGA5^HMA2^, RGA4/RGA5, RGA4/RGA5^HMA2^, RGA4/RGA5/AvrPib, RGA4/RGA5^HMA2^/AVR-Pia, AVR-Pia, and AvrPib. The *N. benthamiana* leaves were infiltrated with a single strain or combinational strains of *A. tumefaciens* that expressed individual proteins, and images were taken 3 d after infiltrations under the visible and ultraviolet lights. (*Middle*) Cell death intensity was scored as an hypersensitive reaction (HR) index based on representative pictures for different values of HR indices in *SI Appendix*, Fig. S4. Each sample represented three biological replicates, and the total number of repeats was 60. Each of the three biological replicates in different colors was labeled in box plots. Differences among the samples were assessed by Tukey’s honestly significant difference test (*P* < 0.01). The LUC activity of rice protoplasts was determined at 16 h after transfection, with the empty vector as the control or vector combinations. Average values and SDs were calculated from three independent experiments. Asterisks indicate that the LUC activity in the individual, vector-transfected rice protoplasts was significantly different from that of the empty vector sample (*P* < 0.05) in Dunnett’s test.

It is known that RGA4, the helper NLR of the RGA4/RGA5 pair, can trigger plant cell death upon the recognition of corresponding MAX effector AVR-Pia or AVR1-CO39 by the sensor NLR RGA5 (26). To verify whether the recognition of AvrPib by RGA5^HMA2^ can activate the RGA4-triggered plant cell death, these two proteins were coexpressed in *N. benthamiana* leaves with RGA4. The coexpression of RGA4/RGA5^HMA2^/AvrPib in *N. benthamiana* leaves induced similar plant cell death as RGA4 or RGA4/RGA5/AVR-Pia, whereas RGA4/RGA5^HMA2^/AVR-Pia, RGA4/RGA5/AvrPib, RGA4/RGA5^HMA2^, RGA5^HMA2^, or AvrPib did not trigger the plant cell death (Fig. 4 and *SI Appendix*, Figs. S4 and S5). The data also showed that AvrPib, but not AVR-Pia, abolished the suppression of RGA5^HMA2^ and triggered the RGA4-mediated cell death. Furthermore, we also assayed the luciferase (LUC) reporter activity of different combinations in the protoplasts of *Oryza sativa* cultivar (cv.) Nipponbare. Similar to the expression of a single RGA4 and the coexpression of RGA4/RGA5/AVR-Pia, the coexpression of RGA5^HMA2^/RGA4/AvrPib resulted in a significant reduction in the LUC reporter activity (Fig. 4*B*), which was consistent with the data obtained from the in planta expression assays in *N. benthamiana* leaves. However, the expression of RGA4/RGA5/AvrPib, RGA4/RGA5^HMA2^/AVR-Pia, RGA5^HMA2^, RGA5^HMA2^/RGA4, or AvrPib in the rice protoplasts could not cause an obvious response (Fig. 4*B*). The above data support that the designed RGA5^HMA2^ can specifically recognize AvrPib in *N. benthamina* and *O. sativa* to mediate the RGA4-triggered cell death (Fig. 1*A* and *SI Appendix*, Table S1).

### RGA5^HMA2^ Confer the RGA4-Triggered Resistance in Transgenic Rice to the Blast Fungus–Carrying AvrPib.

**Fig. 5.**
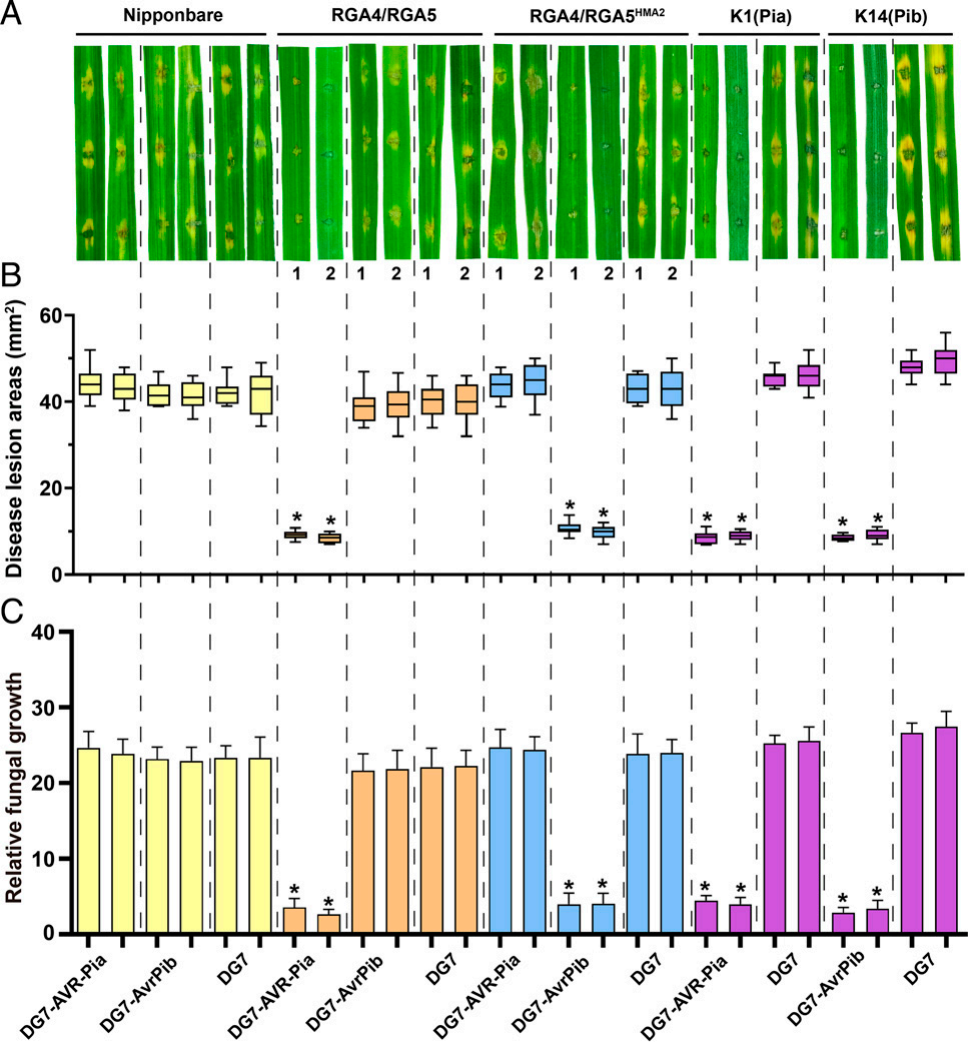
*RGA*4/*RGA*5*^HMA^*^2^ confers specific resistance in transgenic rice to the blast fungus carrying AvrPib. (*A*) Transgenic lines of Nipponbare-expressing *RGA4/RGA5^HMA2^* and the monogenic Lijiangxintuanheigu (LTH) line K14 (carrying *Pib*) form resistant lesions after infection only by the transgenic blast fungus strain DG7-AvrPib but not by DG7 and DG7-AVR-Pia. Similarly, transgenic Nipponbare lines of *RGA4/RGA5* and the LTH K1 line (carrying *Pia*) form resistant lesions only by the blast fungus strain DG7-AVR-Pia but not by DG7 and DG7-AvrPib. In contrast, Nipponbare develops susceptible lesions after infection by all three strains. *RGA5* and *RGA5^HMA2^* were independently cotransformed with *RGA4* into Nipponbare. The T1 generation seedlings were used for inoculation. Inoculation was performed by spotting three 10-μL droplet of conidial suspension (10^5^ conidia/mL) onto the detached leaves of 4-wk-old rice seedlings. Images of two representative leaves of different lines were taken 4 d after inoculation. The numbers 1 and 2 represent two independent transgenic rice lines. (*B*) Box-and-whisker plots show lesion areas on the infected rice leaves from different rice lines in *A* inoculated with different isolates. Each sample from two lines was conducted with three independent biological replicates. Disease areas of each lesion were measured with ImageJ after 4 d of inoculation. The statistical analysis was conducted using an estimation method. Means along with SD were calculated from at least nine lesions of three independent seedlings for each rice line. (*C*) Biomass of the rice blast fungus *M. oryzae* *MoPot*2 in relation to the rice ubiquitin gene. Relative fungal growth was calculated as a ratio (*MoPot*2/*OsUbq*) to reflect the amplification efficiency. The transgenic blast fungus strains were labeled on the bottom. Asterisks represent statistically significant differences in the expression levels of *MoPot*2 at *P* < 0.05. Significant differences were determined using *t* test.

To verify whether the designer NLR receptor RGA5^HMA2^ can confer the RGA4-mediated resistance to the blast disease, the gene pairs of *RGA4/RGA5* and *RGA4/RGA5^HMA2^* were transformed into *O. sativa* cv. Nipponbare, a Geng (Japonica) rice cultivar lacking *Pia* or *Pib*. For each of the gene pairs, five independent transgenic rice lines were generated. qPCR analysis confirmed that the gene pairs were correctly expressed in the transgenic rice lines (*SI Appendix*, Fig. S7*A*). Meanwhile, *AVR-Pia* and *AvrPib* were separately introduced into a field isolate of *M. oryzae* DG7 that is virulent on Nipponbare and the two monogenic lines K1 (with only *Pia*) and K14 (with only *Pib*) of another *O. sativa* cv. Lijiangxintuanheigu (30) (Fig. 5). Pathogenicity assays confirmed that the DG7 transformants carrying either *AVR-Pia* or *AvrPib* were still virulent on Nipponbare but became avirulent on K1 or K14, respectively (Fig. 5). Furthermore, we evaluated the response of transgenic rice leaves (T1 generation) to the two DG7 transformants through wound inoculation. As expected, all the transgenic rice lines expressing *RGA4/RGA5* formed confined, necrotic lesions only after infection by the DG7 transformants with *AVR-Pia*, indicating that they were resistant to the *M. oryzae* isolates with *AVR-Pia*. In contrast, the *RGA4/RGA5^HMA2^* transgenic rice lines became resistant only to the DG7 transformants carrying *AvrPib* but not to those with *AVR-Pia* (Fig. 5). qPCR analysis showed that the effector genes were correctly expressed in all *M. oryzae* transformants infecting incompatible and compatible cultivars (*SI Appendix*, Fig. S7*B*). These results together demonstrated that engineered *RGA5^HMA2^* could confer the *RGA4*-mediated resistance in transgenic rice, specifically to the blast fungus isolates expressing the *AvrPib*.

## Discussion

Generally, plant individual NLR receptors confer resistance only to pathogens carrying the corresponding Avr effectors but not to pathogens lacking the corresponding Avr effectors (6, 31). Thus, many investigations have been undertaken to explore the ways to develop broad spectrum resistance or multilines with distinct resistance profiles (2–5, 31). A promising approach is to engineer plant target proteins of Avr effectors or the IDs of NLR receptors that determine recognition specificity to pathogens (32, 33). Notably, a number of studies have revealed the structural bases of the recognition between IDs and Avr effectors that may guide the engineering of IDs (10, 26, 27, 34, 35). In fact, there have been several reports showing the successful engineering of the plant target proteins or IDs (9, 12, 13, 35). In particular, a recent study reported that a modified Pik-HMA could recognize AVR-Pik variants (28). In another study, the modified RGA5-HMA variants can perceive AVR-PikD besides AVR-Pia and AVR1-CO39, resulting in the activation of immune responses in *N. benthamiana* but not in the transgenic rice (35). These studies based on prior knowledge of molecular or structural biology established the conceptual framework that the Avr effector–targeted plant proteins and IDs of NLR receptors can be engineered to confer new types of resistance. However, a report is yet lacking on how to generate an ID that gains the capacity to recognize noncorresponding effectors whose direct plant target proteins are unknown.

In this study, we generated an ID RGA5-HMA2 that can recognize noncorresponding AvrPib from the rice blast fungus, based on the high structural similarity between AvrPib and the RGA5-corresponding effectors AVR-Pia and AVR1-CO39 (20, 25–27). Although AvrPib has been determined as an MAX effector, and its cognate NLR receptor Pib-encoding gene has been cloned, it remains unknown how AvrPib triggers Pib-mediated resistance (20, 22). Pib is a CC-NLR receptor that lacks the HMA domain (36, 37). Thus, it was immensely challenging to design RGA5-HMA2, which had involved multiple steps of rational mutagenesis on the RGA5-HMA domain. The first step was to compare the structures of AvrPib and AVR1-CO39 for predicting the potential interface in AvrPib, whereby we found that the R23 and V27 residues in AvrPib may correspond to the V36 and Y40 sites of AVR1-CO39 that are located at the interface binding to RGA5-HMA (Fig. 1*B* and *SI Appendix*, Fig. S6). Accordingly, we made the two substitutions, S1027V and G1009D, in the interface of the RGA5-HMA domain (Fig. 1*B*). However, this mutation failed to make the variant RGA5-HMA^S1027V, G1009D^ interacting with AvrPib. Then, we made an RGA5-HMA^K/E^ variant by substituting all the K residues in the K-rich region of the RGA5-HMA variable at its C terminus, which presumably might have blocked the interaction between RGA5-HMA and AvrPib, since AvrPib has a positively charged patch on the surface (Fig. 2*A*) (20). Again, the RGA5-HMA^K/E^ variant was unable to interact with AvrPib. Finally, we contrived the third variant RGA5-HMA2 by combining all the above mutations, which resembled the native RGA5-HMA in structure and gained the AvrPib-binding capacity. This study also confirmed that RGA5^HMA2^, a designer RGA5 NLR receptor integrated with the engineered HMA domain (RGA5-HMA2), had the ability to recognize AvrPib, a noncorresponding effector, to induce RGA4-triggered immunity in *N. benthamiana* and rice (Figs. 4*A* and 5 and *SI Appendix*, Table S1). However, RGA5^HMA2^ lost the capacity to recognize the matching effector AVR-Pia, different from Pikp-HMA, that not only retained the original binding capability but also expanded recognition profiles (28). Therefore, future studies are required to investigate whether RGA5-HMA2 can be further engineered to expand the recognition profiles.

This study, in particular, demonstrated that transgenic rice expressing full-length RGA5^HMA2^ confers resistance to the rice blast strains expressing AvrPib, implying that the IDs of the rice NLR receptors can be engineered to confer rice resistance to the blast fungus isolates with distinct types of effector proteins, including conserved effectors. As previously reported, *M. oryzae* carry diverse MAX effectors, some of which are isolate specific, acting as Avr effectors, while others are conserved in all isolates (18). The targeted engineering of the HMA domain for binding to the specific effectors may generate a series of designer NLR receptors with distinct recognition specificities, which will be useful for efficiently breeding blast-resistant rice multilines harboring the same genetic background but distinct resistance profiles. The rational deployment of such multilines will prevent the erosion of the single *R* gene–mediated resistance and possibly minimize the risk of the emergence of virulence races (2, 3, 38, 39). Our results also have a substantial impact on the targeted engineering of the HMA domain for binding to conserved effectors, by which we may generate designer NLR receptors that can confer broad race spectrum resistance.

In summary, our findings demonstrate that different types of rice NLR receptors with HMA domain can be generated by engineering the ID to confer resistance to the *M. oryzae* isolates carrying noncorresponding MAX effectors without knowing their direct plant targets and provide a practical approach for efficiently breeding rice multilines and a strategy for generating broad race spectrum resistance to the blast disease.

## Materials and Methods

### In Vitro and In Vivo Expression Constructs.

*RGA5-HMA* (from nucleotides 2,944 to 3,348) was amplified from *RGA5* (full length), and RGA5-HMA2 mutants were generated by using the fast multisite mutagenesis kit (Transgen). For the pull-down assay, *RGA5*-*HMA* and mutants thereof were cloned into pETMBP1a with HA-MBP-tag, and *AVR*-*Pia* and *AvrPib* were inserted into pHAT_2_ (kindly supplied by Arie Geerlof, Helmholtz Zentrum München, München, Germany), with HA-tag in their N-termini. For the Y2H assay, *RGA5*-*HMA* and its mutants were ligated into pGADT7 (Clontech) while *AVR-Pia* and *AvrPib* were independently inserted into pGBKT7 (Clontech). For the transient expression in *N. benthamiana*, the full-length of RGA5, RGA5^HMA2^, RGA4, *AVR*-*Pia*, and *AvrPib* were individually cloned into the vector pCAMBIA 1,305 with FLAG-, HA-, or GFP-tag. For the expression in *O. sativa*, *RGA5*, *RGA5^HMA2^*, *LUC*, *AVR*-*Pia*, and *AvrPib* were inserted into pUC19. For generating the transgenic strains of *M. oryzae*, *AVR*-*Pia* and *AvrPib* with their 1.1-kb native promoters and 0.3-kb 3′-downstream regions were individually amplified and cloned into the vector pKN (40). For generating transgenic rice, the full length of *RGA5* and *RGA5^HMA2^* were inserted into the vector pCAMBIA1305 with their native promoters, while *RGA4* was ligated into pCAMBIA1300 with its native promoter. All the primers used in this study are listed in *SI Appendix*, Table S2.

### Fungal Strains and Rice Transformation.

The *M. oryzae* strain DG7 was routinely maintained on the oatmeal tomato agar (OTA) plates (20, 40). The DG7 protoplasts were transformed using the linearized pKN vectors carrying *AVR*-*Pia* or *AvrPib*, as described previously (41). Positive transformants were selected with geneticin (Invitrogen). DG7 and transformants (DG7-AVR-Pia and DG7-AvrPib) were cultured on OTA plates at 26 °C for conidiation (40). For rice transformation, rice callus was induced from embryos of mature seeds of cv. *Nipponbare* (no *Pia* gene). Rice transformation was conducted using the *Agrobacterium tumefaciens*–mediated method, as described previously (42).

### For the Virulence Assay of *M. oryzae* Strains.

The top leaves of 4-wk-old seedlings of *Nipponbare*, K1, K14, and the transgenic rice lines were wound inoculated with the conidial suspensions at a concentration of 10^5^ conidia/mL in 0.025% Tween-20, as described in ref. 43. Inoculated leaves were incubated in a moist, dark chamber at 26 °C for 36 h and then with a 12-h light/12-h dark cycle for additional 4 d. Images of typical disease lesions formed on the leaves were recorded after 7 d. All the assays were repeated at least thrice.

### qPCR Assays and Pathogenicity Analysis.

To measure the expression level of effectors and *NLR* receptors, the inoculated rice leaves were harvested from the transgenic rice infected with different *M. oryzae* transformants. The total RNA of 4-d infected leaves was isolated with the RNA extraction kit (Vazyme) and reverse transcribed into complementary DNA (cDNA) with the HiScript First Strand cDNA Synthesis Kit (Vazyme). qPCR was performed on an ABI Quantstudio 6 Flex (Thermo Fisher Scientific). For the statistical analysis of *M. oryzae* pathogenicity, lesion areas on nine infected rice leaves from different rice lines were measured with ImageJ (https://imagej.net/).

### Yeast Two-Hybrid Assays.

The Yeastmaker yeast transformation system (Clontech) was used to assay protein–protein interactions. The coding sequences of *AvrPib* and *AVR*-*Pia* without the signal peptide were inserted into the plasmid pGBKT7 as the bait vectors, and the coding sequences of *RGA5* and its mutants were cloned into the plasmid pGADT7 as the prey vectors. The prey vectors and the corresponding bait vectors were cotransformed into the yeast strain Y2H *AH109*, following the manufacturer’s instructions. The transformants were cultured on SD/-Trp/-Leu plates and SD/-Trp/-Leu/-His plates with 3-amino-1,2,4-triazole for 3 d at 30 °C.

### Transient Protein Expression and Cell Death Assays in *N. benthamiana*.

Transient protein expression in *N. benthamiana* was performed as reported previously (25). The constructs were independently transformed into the *A. tumefaciens* strain *GV3101*, and the resulting transformants were grown in Luria–Bertani medium with 20 μg/mL rifampicin and 100 μg/mL kanamycin at 28 °C for 24 h. The cultures were harvested by centrifugation at optical density 600 (OD_600_) to ∼2 and resuspended with equivalent infiltration buffer (10 mM MES pH 5.6, 10 mM Mgcl_2_, and 150 μM acetosyringone). For the infiltration, OD_600_ for each of the four GV3101 strains containing *P19*, *RGA4*, *RGA5^HMA^* or *RGA5^HMA2^*, and *AvrPia* or *AvrPib*, was adjusted to 0.5 and mixed in the same ratio, giving a final OD_600_ at 2.0. When one or more strains were absent, the final OD_600_ was adjusted to 2.0 with an equal amount of the empty vector strain. The strain mixtures were incubated for 3 h at room temperature prior to infiltration. The infiltrated plants were set in the dark at room temperature, and the cell death of the leaves was visualized and recorded after 3 d under visible and ultraviolet lights (44). The area of lesion size was measured with ImageJ, and HR indices were scored according to the scale modified to range from 0 (no visible necrosis) to 7 (fully necrosis) in *SI Appendix*, Fig. S4, as described previously (45–48).

### Co-IP and Immunoblotting.

The *N. benthamiana* leaf proteins were extracted and detected as described previously (49). In brief, the soluble proteins were extracted by grinding the tissue in liquid nitrogen from 1 g of the *N. benthamiana* leaves infiltrated with the *GV3101* strains with 2 mL extraction buffer (10% glycerol, 25 mM Tris·HCl pH 7.5, 1 mM EDTA, 150 mM NaCl, 2% polyvinylpolypyrolidone [PVPP], 5 mM DTT, 1× protease inhibitor, and 1 mM PMSF). After removing tissue residues by centrifugation, 1 mL supernatant was applied for anti-GFP beads, incubating with gentle rotation for 3 h at 4 °C. The incubated beads were washed three times with the protein extraction buffer without PVPP, and then, proteins were eluted with 50 μL 1× loading buffer. Finally, the samples were boiled for 10 min, followed by separation on 10% SDS–polyacrylamide gel electrophoresis (PAGE) gels and transferred onto PVDF membrane (Millipore) for immunoblotting. The membranes, after blocking overnight with 5% skimmed milk powder, were sequentially probed with a mouse primary antibody against HA- or GFP-tag at a 1:5,000 dilution for 1 h at 37 °C and then with a secondary antibody (anti-rabbit IgG-peroxidase antibody; Sigma-Aldich) at a 1:5,000 dilution. The membranes were washed in Tris-buffered saline containing 0.05% (v/v) Tween 20 buffer (20 mM Tris, pH 8.0, 150 mM NaCl, and 0.05% Tween-20) three times after each incubation with the antibody, and proteins were detected by electrochemiluminescence (Millipore).

### Pull-Down Assay.

The expression and extraction of MBP-RGA5-HMA2, MBP-RGA5-HMA, HA-AVR-Pia, and HA-AvrPib proteins from *E. coli* were performed as described previously (20, 27). Briefly, recombinant protein extracts were stored in binding buffer (20 mM Tris, 150 mM NaCl, 5 mM DTT, 4 mM EDTA, pH 7.4, and 1% Triton X-100). Anti-MBP beads (50 μL) were washed three times in the binding buffer. One milliliter of the binding buffer and 10 μg proteins were separately added to anti-MBP beads and incubated at 4 °C for 3 h with constant rotation. The pellets were washed five times with the binding buffer and boiled for 10 min. After SDS-PAGE gel separation, the proteins were transferred onto the PVDF membrane (Millipore) for immunoblotting and subsequent detection by the anti-MBP antibody.

### MST.

The fluorescent dye NT-647 (MO-L001, NanoTemper Technologies) was used to labeled RGA5-HMA or RGA5-HMA2. The labeled proteins were eluted with the reaction buffer (20 mM phosphate-buffered saline, 150 mM NaCl, and 0.05% [volume/volume] Tween 20, pH 7.4), and mixed with different concentrations of effectors (AVR-Pia and AvrPib) before loading to Monolith NT.115 (NanoTemper Technologies). Finally, the *K*_d_ values of effector and HMA were measured with 30% light-emitting diode power, and data were treated by the KD Fit function of the Nano Temper Analysis Software (version 1.5.41). Each experiment was repeated three times.

### Cell Death Assay of Rice Protoplast.

Rice plants were grown as described previously (50), and protoplasts from the rice leaves were prepared as reported (25). The constructs in various combinations were mixed with empty vector and LUC plasmid and used to transfect rice leaf protoplasts by the polyethylene glycol (PEG) method (51). After 16 h transfection, the LUC activity of the rice protoplasts was measured by the LUC assay system (Promega). The result was compared with negative control (empty vector–transfected protoplasts).

### Expression and Crystallization of the RGA5-HMA2 Domain.

The expression and purification of RGA5-HMA2 (residues 982 to 1,116) were conducted as described previously (52). Crystals of RGA5-HMA2 were grown at 291 K by sitting drop vapor diffusion. The best crystals of the mutant were obtained from the same condition (0.2 M ammonium nitrate and 20% [weight/volume] PEG 3350) and appeared after 5 d. Crystals were harvested into a reservoir buffer containing 20% (volume/volume) glycerol as cryoprotectant and frozen in liquid nitrogen prior to X-ray data collection.

### Data Collection, Structure Determination, and Refinement.

X-ray diffraction data from the crystals of the mutants were collected at a wavelength of 0.97 Å on beamline BL17 and BL19 at the Shanghai Synchrotron Radiation facility. Data were integrated and scaled with the HKL-2000 processing package (53). Structures were solved by molecular replacement using Phaser with 5ZNE as a search model (54). Structures were improved by rebuilding amino acids into the electron density using Coot (54) and further refined using PHENIX with Translation/Libration/Screw restraints (55). The data collection and refinement statistics are shown in Table 1.

**Table 1.** Data collection and refinement statistics

	HMA2
Data collection	
Beamline	SSRF BL17U1
Wavelength (Å)	0.9792
Resolution range (Å)	35.85 to 2.45 (2.53 to 2.45)*
Space group	P 1 2 1
Unit cell	119.03 78.23 121.00 90.00 90.54 90.00
Total reflections	506674
Unique reflections	79,835 (7,656)
Multiplicity	6.7 (6.4)
Completeness (%)	96.78 (93.55)
Mean I/sigma(I)	29.41(2.1)
Refinement Wilson B-factor	62.03
Rmerge^†^	0.083 (0.76)
Rmeas	0.091 (0.82)
CC1/2	0.99 (0.92)
R-work^‡^	0.21 (0.31)
R-free	0.25 (0.35)
Number of nonhydrogen atoms	7,718
Macromolecules	7,672
Protein residues	1,022
RMSDBond lengths (Å)	0.009
Bond angle (°)	1.28
Ramachandran plot (%)^§^	
Ramachandran favored	97.99
Ramachandran allowed	2.01
Ramachandran outliers	0.00
Rotamer outliers	0.00
Clashscore	11.69
Average B-factor	84.11
macromolecules	84.20
Number of TLS groups	71

* Numbers in parenthesis are for the highest resolution data shell.

^†^ *R_merge_* = Σ*_hkl_*Σ*_i_*(|*I_i_*(*hkl*) −〈*I*(*hkl*)〉|)/Σ*_hkl_*Σ*_i_I_i_*(*hkl*).

^‡^ *R_work_* = Σ*_hkl_(ǁF_obs_|*–*|F_calc_*|*|)*/Σ*I_hkl_|F_obs_*|.

^§^ As evaluated by MolProbity.

## Data Availability

X-ray structure data have been deposited in Protein Data Bank (7DV8). All other study data are included in the article and/or *SI Appendix*.
